# Authors' Response to Peer Reviews of “COVID-19 Return to Sport: NFL Injury Prevalence Analysis”

**DOI:** 10.2196/38755

**Published:** 2022-04-22

**Authors:** Troy B Puga, Joshua Schafer, Prince N Agbedanu, Kevin Treffer

**Affiliations:** 1 College of Osteopathic Medicine Kansas City University Kansas City, MO United States; 2 School of Medicine University of Kansas Kansas City, MO United States; 3 Department of Health Sciences Division of Science, Technology, Engineering, and Math Friends University Wichita, KS United States; 4 Department of Osteopathic Manipulative Medicine College of Osteopathic Medicine Kansas City University Kansas City, MO United States

**Keywords:** COVID-19, sport, injury, prevalence, cause, data, statistics, pain, training, practice, physiology, adaptation

*This is the authors’ responses to peer-review reports of “COVID-19 Return to Sport: NFL Injury Prevalence Analysis”*

## Round 1

Thank you for reviewing our paper [1]. We have provided response in regard to all editorial and reviewer comments.

### Reviewer D [2]

#### General Comments

Thank you for your comments on our paper and for taking the time to review our research.

#### Specific Comments

1. Thank you for your comment. The data used in this study are publicly available data. As defined by United States Department of Health and Human Services policy 45 CFR 46.102, publicly available data do not qualify as human subjects research and do not require institutional review board approval. However, the authors of this research strive to uphold all principles of ethical research and principles of medical ethics.
2. Thank you for your comment; we have included in the introduction and methods that contact injuries are included in this study, as football is a contact sport, making contact a nonmodifiable risk factor.

### Anonymous [3]

#### General Comments

 Thank you for your comments and taking the time to review our paper. We have addressed the hours as a limitation, and we do not have access to the individuals or their specific training hours as we used a public data set. We also addressed the fact that there would be potential recall bias, as the research goes several years into the past and may not provide an accurate number of hours per year if this were to be undertaken. We also have added support that there was a reduction in training among the majority of athletes across all levels of sport due to lockdown restrictions to mitigate the spread of COVID-19. We have also provided support that the National Football League training facilities were shut down between March 25, 2020, and May 19, 2020. We expanded on the conclusion as we have evidence of reduction in training across all levels of sport and facility closures due to COVID-19 precautions.

#### Specific Comments

1. Thank you for your comment. We have defined resistance exercise and changed the wording of “post-resistance exercise” to “after resistance exercise” in order to provide a clearer description.
2. Thank you for your comment, we have removed the cited figure from the abstract.
3. Thank you for your comment. We have provided rationale within the methods section as to why sick days were not included. Sick days were not included due to the fact that literature from other sports analyses have stated that it is best to report incidence of illness separate from injuries. In addition, sick days would not accurately represent the possibility of increased injuries due to less training and could potentially create a confounding variable.

## Abbreviations

CHERRIES: Checklist for Reporting Results of Internet E-Surveys
